# CRISPR‐Enhanced Hydrogel Microparticles for Multiplexed Detection of Nucleic Acids

**DOI:** 10.1002/advs.202206872

**Published:** 2023-02-01

**Authors:** Yoon Ho Roh, Chang Yeol Lee, Sujin Lee, Hyunho Kim, Amy Ly, Cesar M. Castro, Jinwoo Cheon, Jae‐Hyun Lee, Hakho Lee

**Affiliations:** ^1^ Institute for Basic Science (IBS) Center for Nanomedicine Seoul 03722 Republic of Korea; ^2^ Graduate Program of Nano Biomedical Engineering (NanoBME) Advanced Science Institute Yonsei University Seoul 03722 Republic of Korea; ^3^ Center for Systems Biology Massachusetts General Hospital Research Institute Boston MA 02114 USA; ^4^ Department of Radiology Massachusetts General Hospital Harvard Medical School Boston MA 02114 USA; ^5^ Department of Pathology Massachusetts General Hospital Harvard Medical School Boston MA 02114 USA; ^6^ Department of Medicine Massachusetts General Hospital Harvard Medical School Boston MA 02114 USA; ^7^ Department of Chemistry Yonsei University Seoul 03722 Republic of Korea

**Keywords:** CRISPR/Cas, human papillomavirus, hydrogel microparticles, isothermal amplification, multiplexed assays

## Abstract

CRISPR/Cas systems offer a powerful sensing mechanism to transduce sequence‐specific information into amplified analytical signals. However, performing multiplexed CRISPR/Cas assays remains challenging and often requires complex approaches for multiplexed assays. Here, a hydrogel‐based CRISPR/Cas12 system termed CLAMP (*C*as‐*L*oaded *A*nnotated *M*icro‐Particles) is described. The approach compartmentalizes the CRISPR/Cas reaction in spatially‐encoded hydrogel microparticles (HMPs). Each HMP is identifiable by its face code and becomes fluorescent when target DNA is present. The assay is further streamlined by capturing HMPs inside a microfluidic device; the captured particles are then automatically recognized by a machine‐learning algorithm. The CLAMP assay is fast, highly sensitive (attomolar detection limits with preamplification), and capable of multiplexing in a single‐pot assay. As a proof‐of‐concept clinical application, CLAMP is applied to detect nucleic acid targets of human papillomavirus in cervical brushing samples.

## Graphical abstract

A new strategy is demonstrated for multiplexed CRISPR/Cas assays. In this approach, each Cas reaction takes place inside a spatially‐encoded hydrogel microparticle (HMP). A machine‐learning (ML) algorithm then identifies coded HMPs and quantifies their intended molecular targets. The method is scalable to detect many targets in a single‐pot format, facilitating high‐throughput diagnoses.

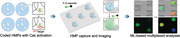

## 1 Introduction

Nucleic acids (NAs) are essential biomarkers for disease detection and monitoring, commonly detected through a quantitative polymerase chain reaction (qPCR). Recent advances in gene editing have introduced clustered regularly interspaced short palindromic repeats (CRISPR) technologies as a powerful mechanism for NA detection.^[^ ^1^, ^2^ ^]^ One unique advantage of the CRISPR system is the trans‐cleavage activity found in CRISPR‐associated (Cas) protein subfamilies. When a Cas protein recognizes its target NA via guide RNA (gRNA), the resulting ternary structure becomes an active endonuclease, indiscriminately degrading single‐stranded NAs.^[^ ^3^ ^]^ This novel property allows Cas complexes to amplify an analytical signal with precision down to single nucleotide variants — only activated Cas complexes can trigger the signal generation by cleaving abundant, non‐targeted signaling probes, for example, single‐stranded NAs with a fluorescent dye (F) and quencher (Q) pair. Indeed, the CRISPR/Cas systems have demonstrated superior sensitivity and specificity in detecting NA targets across diverse sources, including pathogens^[^ ^4^, ^5^, ^6^ ^]^ and cancer cells.^[^ ^7^, ^8^ ^]^ The rapid and isothermal nature of the Cas reaction also facilitates advancing on‐site, point‐of‐care diagnostics.^[^ ^1^, ^9^, ^10^ ^]^

High‐throughput and multiplexed NA detection,^[^ ^11^ ^]^ a key requisite in most molecular diagnostics,^[^ ^12^ ^]^ remains an important unmet need when expanding CRISPR/Cas's reach in biosensing. Technical hurdles, particularly for single‐pot, multi‐target detection, continue to challenge progress. Unlike qPCR wherein different F‐Q probes can be turned on in a target‐specific manner, CRISPR/Cas assays lose sequence specificity due to non‐selective probe cleavage by Cas nucleases.^[^ ^2^ ^]^ This issue has been partly addressed by screening Cas effectors with differential preferences towards cleavage motifs,^[^ ^13^ ^]^ but the availability of orthogonal Cas effectors limits multiplexing to four targets. An appealing alternative is to compartmentalize Cas reactions and run parallel assays. Droplet fluidics have been adopted for such purposes,^[^ ^14^ ^]^ demonstrating high‐throughput NA detection. The droplet approach, however, can be process‐intensive and requires sophisticated tools to generate, coalesce, and detect individual droplets. Another fluidic approach used multiple fluidic chambers to contain target‐specific CRISPR/Cas12 complexes;^[^ ^15^ ^]^ this configuration is essentially a microwell‐type assay, requiring different CRISPR/Cas12 loading per well.

We reasoned that hydrogel microparticles (HMPs) could serve as a CRISPR/Cas reaction container. HMPs are widely used in bioassays:^[^ ^16^, ^17^ ^]^ i) the porous HMP inner structure allows for nearly unhindered motion of biomolecules, leading to solution‐like diffusion and reaction kinetics;^[^ ^18^ ^]^ ii) HMPs’ non‐fouling nature suppresses nonspecific target binding in complex matrices;^[^ ^19^, ^20^ ^]^ and iii) monodisperse, coded HMPs can be fabricated through lithographic methods, providing high multiplexing capability.^[^ ^21^, ^22^, ^23^ ^]^ We hypothesized exploiting these advantages in CRISPR/Cas assays: incorporating Cas into HMPs would streamline multiplexed NA detection while preserving Cas's enzymatic activity.

**Scheme 1:**
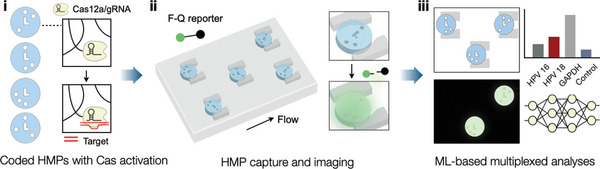
Overview of the multiplexed CLAMP assay. i) Spatially‐coded hydrogel microparticles (HMPs) are loaded with Cas12a/gRNA complexes, with each code denoting a different nucleic acid (NA) target. These particles are mixed with a sample, and Cas12a/gRNA complexes are activated when target NAs are recognized. ii) HMPs are introduced into a fluidic device along with fluorescent‐quencher (F‐Q) reporters. Activated HMPs become fluorescent with F‐Q reporter cleavage. iii) A machine‐learning (ML) algorithm recognizes HMP codes and quantifies particles’ fluorescent intensities, generating assay results on multiple NA targets.

Here, we report an HMP‐based CRISPR/Cas system, termed CLAMP (*C*as‐*L*oaded *A*nnotated *M*icro‐*P*articles), for multiplexed NA detection (**Scheme** **1**). The assay integrated several technical advances. First, we immobilized Cas proteins in spatially‐encoded HMPs, turning each HMP into a discrete vessel for NA detection. Each HMP type, marked with a spatial code, was specific to a unique NA target; upon recognizing its intended NA target, the Cas12a/gRNA complexes cleaved F‐Q probes, turning on fluorescence signals inside HMPs. Second, we designed a microfluidic device to facilitate signal detection. The fluidic device made it easy to locate individual HMPs and, more importantly, allowed us to surround HMPs with immiscible oil to improve the signal‐to‐background contrast. Third, we trained a machine‐learning algorithm to identify the HMP type and quantify its fluorescence intensity. Combining these features into an assay system offered the advantages of i) high sensitivity (attomolar detection limits), ii) simple assay processes with no additional labeling and washing steps needed, and iii) multiplexed NA detection in a single pot. To explore its potential clinical use, we applied CLAMP to detect high‐risk human papillomavirus (HPV) DNA targets within cervical brushing samples.

## 2 Results and Discussion

### 2.1 Characterization of HMPs Functionalized with Cas12a/gRNA

**Figure 1:**
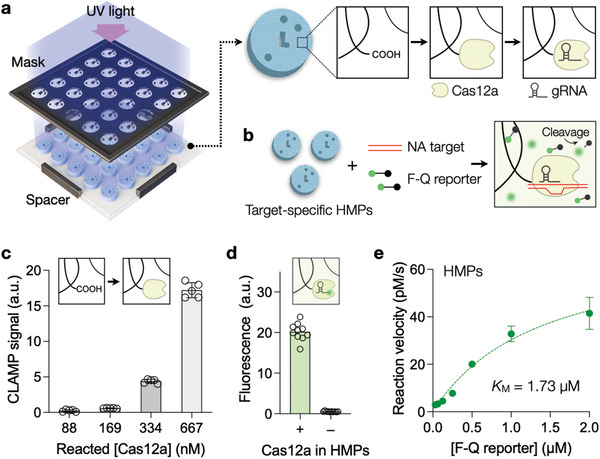
Preparation and characterization of Cas12a/gRNA HMPs. a) Schematic of the HMP synthesis containing Cas12a/gRNA complexes. HMPs were produced by photocuring a precursor solution. UV light was exposed through a photomask to pattern codes in HMPs. Cas12a proteins were immobilized in HMPs by reacting carboxyl groups in the hydrogel with amine groups in Cas12a. Coded HMPs were rendered target‐specific by loading appropriate gRNA. b) Generation of the analytical signal. When the NA target forms a ternary structure with the Cas12a/gRNA complex, the HMP becomes active in cleaving F‐Q reporters, increasing fluorescence. c) Cas12a concentration ([Cas12a]) was varied when reacting with HMPs for loading. The resulting HMPs were used for the CLAMP assay, and the fluorescent intensity was measured. The signal started saturating with [Cas12a] ≥ 667 nM. Bars indicate the mean fluorescent intensity from five replicates. d) Cas12a‐loaded HMPs were reacted with fluorescent gRNA. A high fluorescent signal validated the presence of Cas12a in HMPs. Bars indicate the mean fluorescent intensity from ten HMPs. e) Trans‐cleavage kinetics of Cas12a in HMPs. Reaction velocities were measured at various F‐Q reporter concentrations. The dotted line represents the fit to a Michaelis–Menten model. The enzyme turnover rate was *k*_cat_ = 0.15 s^−1^, and the Michaelis–Menten constant was *K*_M_ = 1.73 µM. The catalytic efficiency (*k*_cat_/*K*_M_) was estimated to be 1.0 × 10^5^ M^−1^ s^−1^. Error bars indicate the SD from ten HMPs.

We prepared HMPs specific to different NA targets (see Experimental Section for details). We first fabricated patterned‐HMPs using mask photolithography (**Figure** **1**a). A prepolymer solution containing acrylic acid, polyethylene glycol diacrylate (PEGDA), and a photoinitiator was exposed to UV through a photomask, which photocured the solution into disk‐shaped particles engraved with identity codes. The optimal concentration of crosslinking monomers (PEGDA) was found to be 5% (volume fraction), which produced structurally robust, highly permeable HMPs.^[^ ^24^ ^]^ To further validate that HMPs had large enough pores, we incubated particles with fluorescent‐dextran whose molecular weight (150 kDa) was similar to that of Cas12a (145 kDa). The partition coefficient (*K*) was then estimated by taking the fluorescent intensity ratio between HMPs and the bulk solution (Figure S1, Supporting Information). The observed *K* was 0.89, indicating HMP's high permeability.

Cas12a proteins were coupled to carboxylic acid groups in HMPs through carbodiimide chemistry that was previously used to immobilize proteins in HMPs.^[^ ^25^ ^]^ Such immobilization was necessary to maintain high Cas12a concentration inside HMPs and prevent inter‐particle crosstalk; without immobilization, proteins would diffuse out of HMPs, leading to signal loss (Figure S2, Supporting Information). Cas12a‐HMPs were then incubated with gRNA, making the particles (Cas12a/gRNA‐HMPs) ready to recognize target NAs. We also prepared the F‐Q reporter probe by conjugating a fluorophore and a quencher at each end of a single‐stranded DNA. This probe generated the fluorescence signal when cleaved by activated Cas12a/gRNA complexes (Figure 1b).

To determine the optimal condition for Cas12a‐HMP preparation, we varied Cas12a concentration [Cas12a] and its conjugation time with HMPs. The prepared Cas12a‐HMPs were then used to detect synthetic NA targets. Both target NAs and fluorescent reporters are diffused into HMPs to react with Cas12a/gRNA complexes.^[^ ^26^ ^]^ The CLAMP fluorescence signal was higher when we used Cas12‐HMPs prepared under higher Cas12a concentration (Figure 1c), but the protein aggregated past the maximal [Cas12a]. Similarly, we found that the optimal reaction time was about 2 h for Cas12a conjugation with HMPs (Figure S3, Supporting Information). The protein binding was stable with negligible leaching of Cas12 proteins from HMPs; compared to the strong CLAMP signal from HMPs, those from supernatants were close to the background during 24‐h incubation in a buffer solution (Figure S4, Supporting Information).

We further characterized Cas12a proteins inside HMPs. The protein amount was first assessed by incubating Cas12a‐HMPs with fluorophore‐labeled gRNA (Figure 1d). The resulting HMP's fluorescence was then referenced to the gRNA calibration curve (Figure S5, Supporting Information). About 5.2 fmol of Cas12a was immobilized per HMP (or 4.9 µM per HMP), assuming that the gRNA binds to the Cas12a in a 1:1 ratio.^[^ ^27^ ^]^ We next evaluated Cas12a‐HMP's catalytic efficiency for trans‐cleavage. We activated Cas12a/gRNA complexes in HMPs by incubating particles with target NAs. Catalytic Cas12a‐HMPs were then mixed with the F‐Q reporter probe, and the fluorescence signal resulting from the probe cleavage was monitored (see Experimental Section for details). Figure 1e shows the initial cleavage rates measured at varying reporter concentrations. Applying the Michaelis–Menten model to the results, we could estimate the enzyme turnover rate (*k*_cat_ = 0.15 s^−1^) and the Michaelis–Menten constant (*K*_M_ = 1.73 µM). The catalytic efficiency (*k*_cat_/*K*_M_) of HMP‐bound Cas12a was 1.0 × 10^5^ M^−1^ s^−1^. This value was in the same order of magnitude as the catalytic efficiency (3.3 × 10^5^ M^−1^ s^−1^) of free Cas12a in solution (Figure S6, Supporting Information)^[^ ^28^ ^]^ but was slightly lower, presumably due to the structural confinement coming from Cas12a immobilization.

### 2.2 CLAMP Assay Optimization

**Figure 2:**
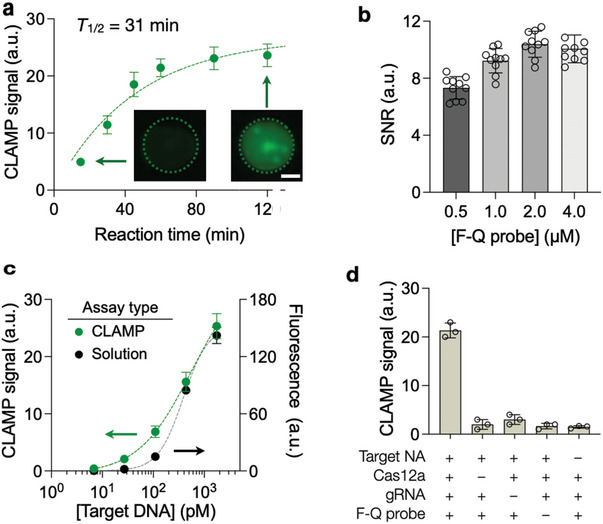
CLAMP assay optimization. a) Optimization of the reaction time between Cas12a/gRNA HMPs and NA targets. The reaction time was varied, and the resulting CLAMP signal was measured. The signal gradually reached a plateau with the estimated half‐time (*T*_1/2_) of 31 min. The reaction time was set to 60 min, which would yield 86% of the final signal. Error bars indicate the standard deviation from ten HMPs. Scale bar, 50 µm. b) Optimization of F‐Q probe concentration. The CLAMP assay was carried out while varying the F‐Q probe concentration ([F‐Q probe]). The signal‐to‐noise ratio (SNR) was estimated as the ratio of fluorescent intensity between targeted and control (no target NA) HMPs. The highest SNR was achieved at [F‐Q probe] = 2 µM. Data are displayed as mean ± sd from ten replicates. c) Serially diluted NA targets were measured by CLAMP (green) and a conventional solution‐based Cas12a assay (black). CLAMP had a lower detection limit (3 pM) than the solution‐based assay (3.6 pM). Error bars are standard deviations from ten HMPs (CLAMP) and three replicates (solution‐based assay). d) Assay validation. The fluorescent signal was observed only when all reaction components were included. Data are displayed as mean ± SD from three replicates.

Using Cas12/gRNA‐HMPs, we established the overall CLAMP assay protocol. We varied the reaction time between Cas12/gRNA‐HMPs and target NAs (**Figure** **2**a) and measured the fluorescence signal. The optimal reaction time was set to 1 hour, at which point the analytical signal started to plateau. The reporter probe's concentration was another factor optimized because it directly influenced the background signal; we selected the probe concentration of 2 µM, which maximized the signal‐to‐noise ratio (Figure 2b). Containing DNA probes inside individual HMPs was also crucial to retaining high sensitivity and minimizing inter‐particle crosstalk. We could achieve such isolation by immersing HMPs in immiscible oil.

Applying the optimal CLAMP protocol, we analyzed serially diluted NA targets (Figure 2c). The analytical detection limit was about 3 pM, and the dynamic range spanned two orders of magnitude. The CLAMP showed higher sensitivity than the solution‐based Cas12a assay. Note that Cas12a concentration was higher in HMPs (4.9 µM) than in solution (0.64 µM), which boosted the trans‐cleavage of F‐Q reporters. We further validated that the CLAMP signal was generated only when all assay components were present (Figure 2d).

### 2.3 CLAMP Detection System for Multiplexing

**Figure 3:**
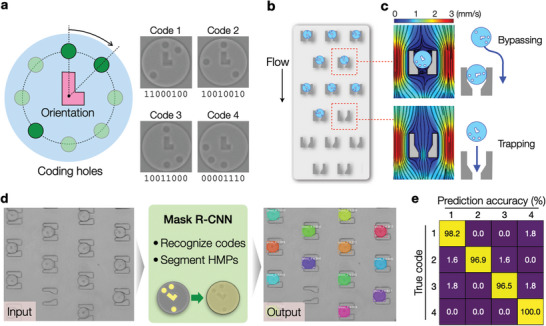
CLAMP engineering for multiplexed target detection. a) Design rules of the identity code in HMPs. The code consisted of an indicator (pink) at the particle center and three holes (green) on the rim. The indicator helped set the internal coordinate to read out the hole patterns. The inset (right) shows four codes used in the current work. b) Schematic of the microfluidic device for HMP capture. An array of traps were laid out to capture individual HMPs in designated locations. c) Simulation of streamlines. Fluidic flow can pass through an empty trap, attracting an HMP (bottom right). Once an HMP is captured, the flow is blocked to divert other HMPs (top right). d) A neural network (Mask R‐CNN) was trained to analyze bright‐field HMP images. The network recognized the identity code in each HMP and then generated a mask for the particle segmentation. e) The confusion matrix demonstrated the high accuracy of the trained network in recognizing the HMP codes.

We engineered the detection system for the multiplexed CLAMP assay. To distinguish target‐specific HMPs, we imprinted particles with spatial codes comprising three dots and an L‐shaped indicator (**Figure** **3**a). The dots were placed among eight possible positions at the HMP's circular edge; the indicator was at the HMP's center to set the readout direction. This rule can generate up to 168 binary codes. For quadruple multiplexing, we chose four equidistant codes; the Hamming distance was the maximal (=4) between codes.

We next designed a microfluidic chip to arrange individual HMPs (Figure 3b). The chip contained an array of HMP‐trapping structures. Each trap had a small rear opening to allow fluid to flow through. Once a particle was trapped, the opening was blocked to increase the flow resistance; other HMPs would then bypass the occupied trap.^[^ ^29^, ^30^ ^]^ We first confirmed the working principle through numerical fluidic simulation (Figure 3c and Figure S7, Supporting Information) and laid out staggered trap columns across the flow direction to minimize channel clogging. The prepared chip demonstrated > 90% trapping efficiency. Importantly, it prevented inter‐particle interaction (i.e., signal bleeding from the direct particle contact), simplified the buffer exchange with oil, and expedited HMP imaging.

We adopted an ML approach to analyze individual HMPs automatically. We selected Mask R‐CNN as an ML engine;^[^ ^31^ ^]^ this neural network can classify objects in an image while simultaneously generating a high‐quality segmentation for each instance (Figure S8, Supporting Information). Our strategy was to use i) the classification to identify HMP types and ii) the segmentation to define a mask for fluorescent detection (Figure 3d). We trained the Mask R‐CNN model with brightfield images of each HMP type (110 images per type). The trained model differentiated 4 different HMP types (Figure 3e) in bright field images, achieving high accuracy (0.979) and *F*_1_ score (0.978) in a separate validation. After HMP identification, the code and the segmentation were transferred to fluorescence images, and the fluorescence intensity of each particle was extracted (see Movie S1, Supporting Information).

### 2.4 CLAMP Assay to Detect Human Papillomavirus DNA

To explore CLAMP's potential for clinical application, we configured CLAMP for multiplexed detection of high‐risk HPV, a leading cause of cervical cancer.^[^ ^32^ ^]^ Cervical cancer incurs high incidence and mortality rates in many resource‐limited countries which often lack early screening infrastructures. We reasoned that the CLAMP system could serve as a portable, rapid diagnostic system to effectively triage suspicious or high‐risk cases.

**Figure 4:**
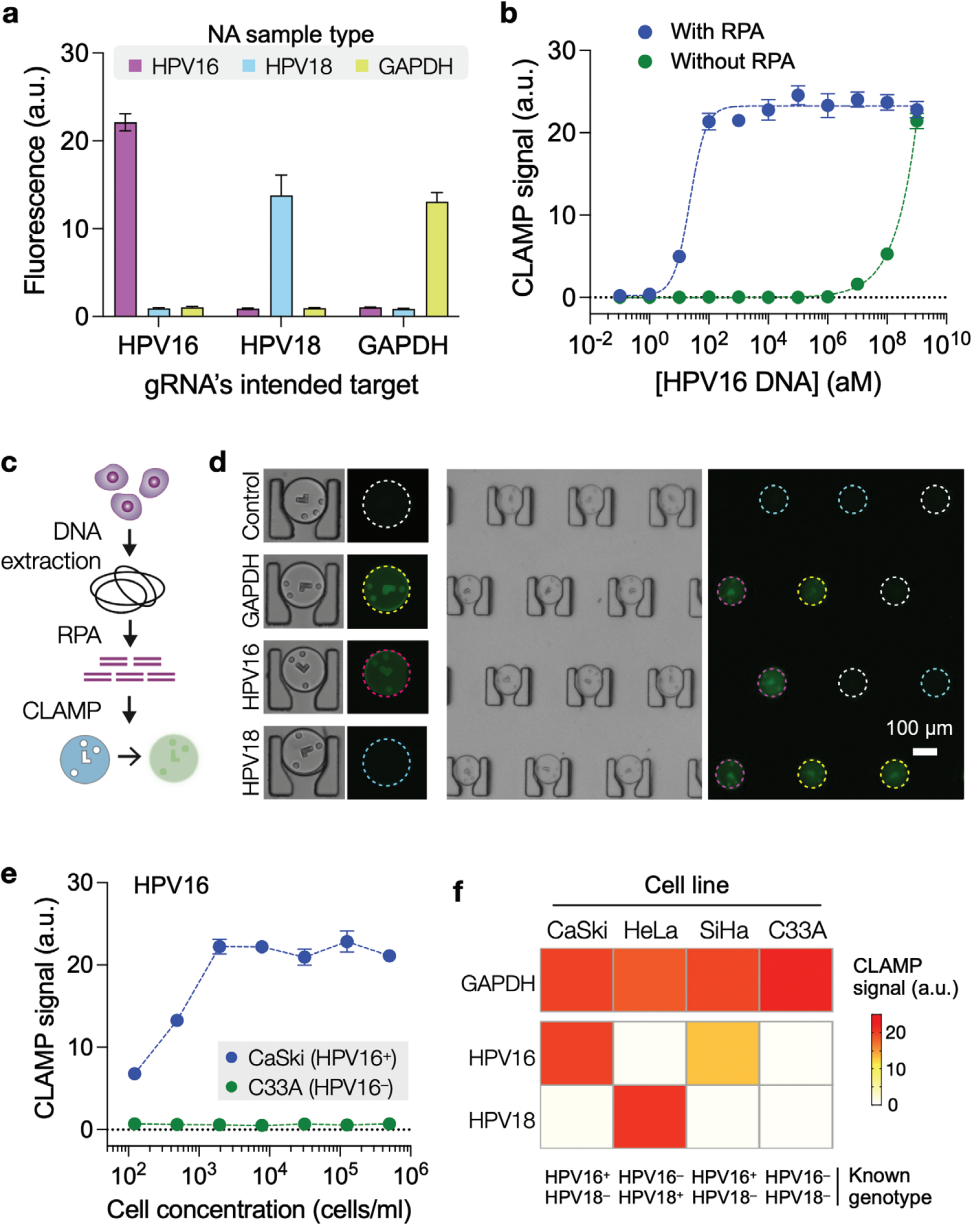
CLAMP assay for multiplexed HPV detection. a) gRNAs were selected for HPV16, HPV18, and GAPDH targets. The selected gRNAs were highly specific only to their intended NA target. The signal was from solution‐based Cas12a assays without RPA. Error bars indicate the standard deviation from three replicates. b) The CLAMP assay analyzed serially diluted synthetic HPV16 DNA samples. Incorporating the RPA step lowered the detection limit to 2 aM (1.2 copy µL^−1^). Data are displayed as mean ± SD from three replicates. c) Schematic of the CLAMP cellular assay. Genomic DNA was extracted and processed by RPA, followed by multiplexed CLAMP detection. d) Example of CLAMP multiplexing. Three types of coded HMPs were rendered to detect GAPDH (yellow circle), HPV16 (pink), and HPV18 (cyan), respectively. HMPs with the fourth code were used as a negative control (white circle). Micrographs show HMPs detecting genomic DNA from HPV16^+^ cells. e) A varying number of cervical cancer cells were analyzed by CLAMP targeting HPV16. With CaSki (HPV16^+^) cells, the detection limit was about 10 cells mL^−1^, whereas the CLAMP signal was negligible with C33A (HPV16^−^) cells. Error bars indicate the standard deviation from ten HMPs. f) The multiplexed CLAMP analyzed four different cell lines (CaSki, HeLa, SiHa, C33A) for HPV16, HPV18, and GAPDH. The CLAMP results matched the known genotype of these cells.

We chose high‐risk HPV subtypes (HPV16, HPV18) as viral targets. Since our goal was to detect HPV genes in human cells, we also added GAPDH as a target for positive control of cellularity. We first designed a set of gRNAs for each target and compared their specificity in the presence of off‐targets (Figure S9, Supporting Information). From this comparison, we selected the gRNA set allowing for multiplexed NA detection in a single pot (**Figure** **4**a). We also added an upstream NA amplification to enhance the overall detection sensitivity. The recombinase polymerase amplification (RPA) method was used because its reaction temperature was close to that of the Cas12a trans‐cleavage assay, allowing for the isothermal reaction for the entire assay. We validated that the optimized RPA primers amplified only targeted genes (Figure S10, Supporting Information). By introducing RPA, we could lower the detection limit to an attomolar range (2 aM; Figure 4b). In comparison, the detection limits were about 145 aM with the RPA alone and about 10 aM by the conventional qPCR (Figure S11, Supporting Information).

We further challenged CLAMP to differentiate a single‐base mismatch. As a model system, we used a non‐modified HPV16 DNA target (matched) and a set of modified HPV16 DNAs with a single‐base mismatch at varying positions (Figure S12a, Supporting Information). The CLAMP assay differentiated the matched target from single‐base mismatched ones (Figure S12b, Supporting Information) when the mismatch was within the seeding region (≈8 bp away from the PAM). The results agreed with the previous report on Cas12a's sequence specificity.^[^ ^33^ ^]^ The PAM‐adjacent region (5–8 bp away from the PAM) forms multiple contacts with several domains of a Cas protein (WED, REC1, and RuvC). A perfect base‐pair match within this region is essential for Cas12a's catalytic activity.

We next applied the CLAMP assay to detect HPV DNA in cervical cancer cells. We extracted genomic DNA (gDNA) from cell lysates, amplified NA targets via RPA, and performed the CLAMP assay (Figure 4c). The assay procedure was close to the clinical HPV test wherein samples (e.g., Pap smear, cervical brushings) are assessed for HPV DNA targets inside cells. To enable multiplexing, we assigned each coded HMP type a different target (Figure 4d). Testing CaSki (HPV16^+^) for HPV16, we observed that the cellular detection limit was about 10 cells mL^−1^ (Figure 4e). In contrast, the signal was negligible with HPV16^−^ C33A cells. We further expanded the multiplexed CLAMP assay to a panel of cervical cancer cell lines. All samples passed the quality check ascertained by the GAPDH signal, and the CLAMP results matched with known HPV16 and HPV genotypes of cells (Figure 4f).

### 2.5 Pilot HPV Detection in Clinical Samples

We then applied the CLAMP assay to detect HPV targets in clinical samples. We obtained excess cervical brushing specimens collected during routine gynecologic evaluation. Samples were selected by a pathologist based on high‐risk HPV status as determined by routine HPV testing (Cobas; Roche, Indianapolis, IN); the test results were blinded until the CLAMP assay was completed. For each given sample, we extracted its gDNA, amplified NA targets (HPV16, HPV18, GAPDH), and performed the multiplexed CLAMP detection (see Experimental Section for details).

**Figure 5:**
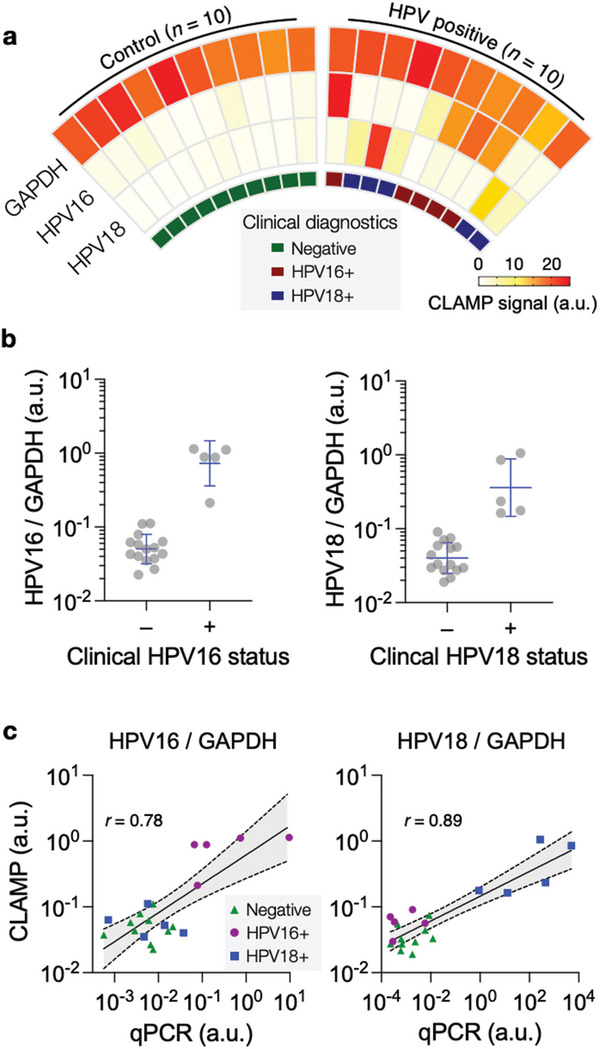
CLAMP testing with clinical samples. a) Cervical brushing samples (*n* = 20) were processed to detect HPV16, HPV18, and GAPDH via CLAMP. All samples showed high GAPDH signals, passing the quality control. The HPV16 and HPV18 status by CLAMP matched with clinical diagnostics by pathology. b) HPV16 and HPV18 levels were normalized against GAPDH. The normalized HPV16 and HPV18 expressions were significantly higher in clinically‐defined marker‐positive samples (*p* < 0.001; unpaired two‐sided *t*‐test). c) The CLAMP results were compared with those by qPCR. Two methods showed a high correlation. *r*, Pearson's coefficient.

**Figure** **5**a summarizes the CLAMP‐HPV results. All samples showed a high enough GAPDH signal to pass quality control (Figure S13, Supporting Information), and no statistical difference was observed between HPV‐positive and negative samples (*p* = 0.74, unpaired two‐sided *t*‐test). Importantly, CLAMP results agreed with conventional pathology‐derived HPV diagnoses. We set the threshold for marker positivity as 3•*σ*, where *σ* is the standard deviation of a blank sample. All marker‐positive samples showed signals higher than the threshold, whereas marker‐negative samples had signals lower than the threshold (Figure 5b). The CLAMP results further matched those by qPCR (Figure 5c), which confirmed the CLAMP's capability for quantification.

## 3 Conclusion

Cas systems are increasingly adopted for biosensing;^[^ ^34^, ^35^, ^36^ ^]^ they offer new ways to amplify analytical signals with precision down to single nucleotide variants. In this work, we encapsulated the Cas assay into biocompatible, coded HMPs. The resulting CLAMP assay brought in unique advantages: i) Cas‐functionalized HMPs could be specific to different NA targets (down to single‐base mismatch) while being identified by unique spatial codes, ii) HMPs locally maintained a high dose of the detection agent (Cas12a/gRNA) to boost the analytical signal, and iii) with Cas12a/gRNA immobilized inside HMPs, each particle can detect its designated NA target. These advantages enabled CLAMP to detect different NA targets (multiplexing) in a single‐pot assay scheme. Furthermore, immobilizing Cas12a/gRNA in HMPs allowed us to concentrate the complex without compromising its catalytic activity, which rendered the CLAMP assay more sensitive than solution‐based Cas assays. These aspects (multiplexing, high sensitivity, scalability) distinguish CLAMPS from other CRISPR/Cas assays (see Table S1, Supporting Information for comparison). The CLAMP assay successfully detected high‐risk HPV targets in the ensuing pilot study, demonstrating a potential for on‐site HPV screening.

Activated Cas proteins cleave reporter probes indiscriminately, making it difficult to perform multiplexed detection. Our solution to this formidable challenge was to transform HMPs into an independent vessel for the Cas reaction; we exploited HMPs’ superior hydrophilicity and compatibility with microfabrication. The fluidic chip for HMP capture was another critical component. Spatially isolating HMPs facilitated their detection via imaging. More importantly, we could confine cleaved fluorescent probes inside HMPs to prevent signal bleed‐through among HMPs. This top‐down approach simplified the assay development. It required only a single pair of a Cas effector and a fluorescent reporter, contrasting with other multiplexing approaches (e.g., multiple Cas effectors and fluorescent reporters).^[^ ^13^, ^14^ ^]^ The CLAMP method is also economically scalable to accommodate many targets. HMPs can be mass‐produced, and the spatial codes are readily expandable to incorporate more binary bits. A major challenge, however, would be designing primer sets for multiplexed amplification. As shown in Figure S9, Supporting Information, primers designed for singleplex assays may fail to work in one‐pot assays due to off‐target amplification, necessitating the process of iterative primer design and screening. To our best knowledge, up to nine targets were amplified in a single pot via multiplexed RPA.^[^ ^15^ ^]^

We expect to further develop CLAMP for use in point‐of‐care diagnostics. First, a compact system could be built for onsite operations. Two functions are required in the CLAMP assay: i) maintaining a constant temperature for the isothermal CLAMP reactions and ii) taking photos of HMPs. A smartphone could be a powerful base platform for such operations. A phone can be programmed to control a simple heater, and, more importantly, it can be coupled with simple optics to enable high‐resolution and sensitive imaging, effectively replacing a bulky microscope.^[^ ^37^, ^38^, ^39^, ^40^, ^41^ ^]^ Indeed, using a smartphone camera‐based system, we could identify HMPs with their codes and measure the fluorescent signals (Figure S14, Supporting Information). Second, we should explore lyophilizing CLAMP reagents to simplify transport logistics and extend shelf life. Previous reports demonstrated that lyophilized Cas proteins and HMPs could be resuscitated after storage in ambient conditions.^[^ ^42^, ^43^ ^]^ We need a similar test on HMPs pre‐conjugated with Cas12/gRNA complexes. Finally, we should consider incorporating DNA extraction into the CLAMP workflow, which will minimize the risk of errors from sample contamination and user intervention. Heat‐based cell lysis is promising^[^ ^44^, ^45^ ^]^ as it can be executed with an existing CLAMP device. With these improvements, we believe the CLAMP assay would be a portable and field‐usable platform that facilitates NA profiling for clinical diagnoses.

## 4 Experimental Section

### Materials

The following items were purchased for NA detection: oligonucleotides (Bioneer, Korea), gBlock gene fragments (Integrated DNA Technologies, USA), TwistAmp Basic kit (TwistDX, UK), EnGen Lba Cas12a (New England Biolabs), Murine RNase inhibitor (New England Biolabs), and NEBuffer 2.1 (New England Biolabs). For HMP preparation, poly(ethylene glycol)diacrylate (PEGDA 700, Mn = 700 Da), poly(ethylene glycol) (PEG 600, Mn = 600 Da), 2‐hydroxy‐2‐methylpropiophenone (Darocur 1173) photoinitiator, acrylic acid, and Tween 20 were used, all purchased from Sigma‐Aldrich (USA). For bioconjugation, N‐hydroxysuccinimide (NHS; 24500) and N‐(3‐(dimethylamino)propyl)‐N′‐ethyl‐carbodiimide hydrochloride (EDC; 22980) from ThermoFisher Scientific (USA) were used. In the fluidic chip operation, polydimethylsiloxane elastomer (PDMS; Sylgard 184, Dow Corning) and hydrofluoroether (HFE‐7500; 3 M USA) were used. DNA sequences used in this study are listed in Table S2, Supporting Information.

### HMP Fabrication and Bioconjugation

An HMP precursor solution was prepared by mixing 5% (v/v) PEGDA 700, 10% (v/v) acrylic acid, 80% (v/v) PEG 600, and 5% (v/v) photoinitiator. The mixture was poured on a glass slide and covered with a photomask (MicroTech, Korea); spacers (height, 52 µm) were placed between the glass slide and the photomask to set the HMP height. The mixture was then exposed to UV light (through the mask, 1200 msec) to polymerize the precursor. Note that the photomask and the glass slide were coated with PDMS, making it easy to detach polymerized HMPs. HMPs were first suspended in an ethyl alcohol solution to remove unreacted precursors and then washed HMPs four times with 1× PBST (1× PBS with 0.05% Tween 20). To prepare HMPs for bioconjugation, HMPs (≈1000 particles in 400 µL 1x PBST) were mixed with NHS (80 µL, 20 mg mL^−1^ in 1× PBST) and EDC (80 µL, 20 mg mL^−1^ in 1× PBST), incubated the mixture on a thermal shaker (21.5 °C, 30 min, agitation at 1500 rpm), and then triple‐rinsed HMPs in 1× PBST. For Cas12a conjugation, the activated HMPs mixed with 0.6 µM Cas12a (1× PBST), allowed the mixture to react (21.5 °C, 2 h, agitation at 1500 rpm), and triple‐washed HMPs with 1× NEBT (1× NEBuffer 2.1 with 0.05% Tween 20). Finally, Cas12a‐immobilized HMPs (Cas12a‐HMPs) were incubated with 1 µM target‐specific gRNA (37 °C, 30 min) and triple‐washed with 1× NEBT. The prepared Cas12a/gRNA‐HMPs were stored at 4 °C for further use.

### HMP Permeability Test

Two types of HMPs with different cross‐linking degrees were prepared by varying the amount of PEGDA in the precursor solution: highly porous HMPs (5% v/v PEGDA 700) and non‐porous HMPs (95% v/v PEGDA 700). To test the HMP permeability, HMPs were contained inside a microfluidic chamber and injected with 150 kDa FITC‐dextran (2.5 mg mL^−1^) at the flow rate of 50 µL h^−1^. Fluorescent images were taken before and after 2 h of FITC‐dextran injection. The partition coefficient (*K* = *I*_gel_/*I*_sol_) was calculated by dividing the background‐subtracted fluorescent intensity of hydrogel (*I*_gel_) by that of bulk solution (*I*_sol_).

### Fabrication of Microfluidic Chips

The device was laid out using design software (AutoCAD, USA) and had the master mold (SU‐8) fabricated by a company (Microfit, Korea). The height of the fluidic channel was 85 µm. To make the fluidic device, a PDMS mixture (10:1 base to curing agent ratio) was prepared, poured over the master mold, and cured the polymer (60 °C, overnight). The cured PDMS slab was then peeled off, cut into the desired size, and punched holes (0.8 mm biopsy punch; Miltex, USA) to define fluidic ports. As a fluidic bottom substrate, a glass slide was separately prepared; PDMS was coated on the slide and partially cured (60 °C, 2 h). The fluidic device was assembled by attaching the PDMS slab to the glass slide. The final device was heat‐cured (60 °C, overnight) to ensure leak‐tight bonding.

### Flow Simulation in Microfluidic Chips

The flow inside the fluidic chip was simulated using Comsol multiphysics 5.6 software (microfluidics module, COMSOL). The two designs were compared, one with an inter‐trap distance of 200 µm and the other with 280 µm. Other design parameters were identical. The fluid was assumed to be water (density, 1000 kg m^−3^; dynamic viscosity, 1 mPa·s) at ambient conditions (20 °C, 101.3 kPa), and the flow speed at the inlet was set to 10 mm s^−1^. A finer mesh and a stationary solver with no‐slip boundary conditions were used. To visualize the flow, 100 streamlines evenly spaced across the fluidic channel were arranged. The HMP trapping was also simulated by introducing a disk (diameter, 140 µm; density 1120 kg m^−3^) inside the fluidic chip.

### Estimating Cas12a Amounts in HMPs

A calibration curve was first generated for the FAM‐labeled gRNA (FAM‐gRNA). A known amount of FAM‐gRNA was injected into the microfluidic device and measured the resulting fluorescent intensity (Figure S5, Supporting Information). The height of the fluidic device was similar to HMP's height to minimize the risk of potential artifacts caused by a height difference. A varying dose of FAM‐gRNA was injected into the microfluidic device and measured the resulting fluorescent intensity (Figure S5, Supporting Information). Cas12a‐HMPs reacted with the excess amount of FAM‐gRNA (37 °C, 30 min) and triple‐washed the HMPs with 1× NEBT. Cas12a/FAM‐gRNA HMPs were then injected into the fluidic device and their fluorescent intensities were measured. The amount of FMA‐gRNA in an HMP was estimated from the calibration curve, assuming that gRNA would bind to Cas12a at a 1:1 ratio.^[^ ^27^ ^]^

### Solution‐Based Cas12a Assay

In optimizing probe sequences and reaction conditions, the solution‐based Cas12a assay was used. A reaction mixture was prepared by combining Cas12a (640 nM), target‐specific gRNA (160 nM), F‐Q probe (2 µM), RNase inhibitor (0.8 U µL^−1^), and target NA in 1× NEBuffer 2.1. The reaction was monitored by measuring fluorescence intensities at 37 °C for 1 h on ViiA 7 Real‐Time PCR System (ThermoFisher Scientific). Note that the fluorescence intensity was corrected by subtracting the background signal measured without target NA.

### Generation of Calibration Curves

A solution‐based assay using F‐Q probes was performed at varying concentrations. The target NA concentration was set to 10 nM in a total volume of 20 µL. After the Cas12a reaction (37 °C, 12 h), the fluorescence intensity of fully cleaved probes (ViiA 7 Real‐Time PCR System) was measured to obtain a calibration curve for solution‐based assays (Figure S15, Supporting Information). The fully cleaved mixture was also injected into the microfluidic device and the resulting fluorescent intensity was measured, generating a calibration curve for the microfluidic device environment.

### Cas12a Reaction in Solution

The reaction mixture was prepared as in the solution‐based assay but the F‐Q probe concentration was varied. The target NA concentration was set to 0.5 nM in a total volume of 20 µL. The Cas12a's trans‐cleavage activity was monitored by measuring the fluorescent intensity for 10 min at every 30 s (37 °C, ViiA 7 Real‐Time PCR System). The initial reaction velocity (*v*) was then obtained by converting the fluorescent intensity to F‐Q probe concentration ([S]). The molar reaction velocities were plotted against F‐Q probe concentrations and fitted to the Michaelis–Menten model, *v* = *E_t_* ·*k*_cat_·[S]/(*K*_M_ + [S]), where *E_t_* is the concentration of catalytic sites (0.5 nM), *k*_cat_ is the enzyme turnover rate, and *K*_M_ is the Michaelis–Menten constant. Prism 9.3 (GraphPad, USA) was used for the fitting.

### Cas12a Reaction in HMPs

Cas12a/gRNA HMPs were prepared and activated by adding 0.5 nM target NA. The activated HMPs were mixed with an F‐Q probe solution, and the mixture was injected into the microfluidic device. The HMPs’ fluorescent intensity for 10 min (37 °C) was monitored and then the initial reaction velocity (*v*) was estimated. The F‐Q calibration was used to convert the fluorescent intensity to the probe concentration ([S]). The data were analyzed to estimate *k*_cat_ and *K*_M_.

### CLAMP Assay

An RPA mix was prepared by combining 240 nM of target‐specific forward and reverse primers, 0.8 U µL^−1^ RNase inhibitor, and target NA. Upon the addition of 14 mM MgOAc, the reaction proceeded at 37 °C for 20 min. The amplified target NAs were then mixed with ≈50 Cas12a/gRNA‐HMPs in 1× NEBuffer 2.1 containing the F‐Q probe (2 µM) and RNase inhibitor (0.8 U µL^−1^). After incubation (1 h, 37 °C), HMPs were introduced to the microfluidic device using manual pipettes or disposable eye droppers. After capturing individual HMPs on‐chip traps, the fluidic chamber was filled with HFE‐7500 oil. After an additional incubation period (30 min, 37 °C), HMPs were imaged using EVOS M7000 (ThermoFisher Scientific) and the image was analyzed with the customized ML algorithm. For a given NA target, the fluorescent signal was measured from about 15 target‐specific HMPs and the mean value was used as an analytical metric. The CLAMP signal was corrected by subtracting the background signal measured without target NA.

### ML Algorithm for Imaging Analyses

For each coded HMP type, 110 HMP images were used for training and additional 20 HMP images for validation. Data were augmented via image rotation and flipping. The Supervisely software (Supervisely OU, Estonia) was used to annotate HMPs and Google Colab to train the ML model (Mark R‐CNN). During the training, the learning rate gradually decreased from 0.01 to 0.0001 in logarithmic steps. Table S3, Supporting Information, lists hyperparameters used in training. The accuracy (*A_c_*) and the *F*_1_ score were calculated according to their standard formulas, *A_c_* = (TP + TN)/(TP + FN + FP + TN) and *F*_1_ = 2·TP/(2·TP + FN + FP), where TP, TN, FN, and FP are true positive, true negative, false negative, and false positive, respectively.

### Cell Culture and NA Extraction

CaSki, SiHa, HeLa, and C33a cells (American Type Culture Collection) were cultured to 80–90% confluency in vendor‐recommended growth media (RPMI‐1640 for CaSki; DMEM for SiHa, HeLa, and C33a) supplemented with 10% fetal bovine serum, 100 U mL^−1^ penicillin, and 100 µg mL^−1^ streptomycin. All cell lines were monitored for mycoplasma contamination using a mycoplasma detection kit (MycoAlert; Lonza, Switzerland). The cells and extracted genomic DNA (gDNA) were extracted using Quick‐DNA Miniprep (Zymo Research, USA) per the manufacturer's instruction. After centrifugation (300 g, 5 min), pelleted cells were suspended in 1 mL genomic lysis buffer (8 min, 20 °C). Cell lysates were then transferred to Zymo‐Spin^TM^ IICR Columns placed in collection tubes, followed by centrifugation (10 000 g, 1 min). The columns were sequentially washed with 200 µL DNA pre‐wash buffer and 500 µL gDNA wash buffer. Cellular gDNA samples were eluted with DEPC‐DW and stored at −20 °C.

### Clinical Sample Analysis

The clinical study was approved by the Partners Healthcare Institutional Review Board (IRB protocol 2019P001372). Informed written consent from all participants was obtained. Cervical brushing during routine gynecologic exams was collected into SurePath liquid‐based Pap test vials (BD, Franklin Lakes, NJ). After the pathologic diagnosis, aliquots of the residual material in the SurePath vials were analyzed in the CLAMP assay. The Quick‐DNA FFPE Miniprep (Zymo Research, USA) was used per the manufacturer's instruction to extract gDNA. Each clinical sample (500 µL) was centrifuged (500 g, 4 min) and the cell pellet was mixed with proteinase K (2 mg mL^−1^) for lysis, followed by heat‐inactivation. The sample was mixed with genomic lysis buffer (700 µL) and isopropanol (200 µL), and the mixture was centrifuged to remove insoluble debris (12 000 g, 1 min). The supernatant was then transferred to Zymo‐Spin^TM^ IICR Columns to capture gDNA via centrifugation (10 000 g, 1 min). Captured gDNA was triple‐washed via centrifugation and eluted with DEPC‐DW. Samples were stored at −20 °C.

### Statistical Analysis

Statistical analyses were performed using GraphPad Prism version 9.5 (GraphPad Software Inc.) or R version 4.2.2. When comparing two groups, a two‐tailed *t*‐test was used. For all statistical tests, *p*‐values < 0.05 were considered significant. Details on data presentation and the same size are specified in figure legends.

## Conflict of Interest

The authors declare no conflict of interest.

## Supporting information

Supporting Information

Supplemental Video 1

## Data Availability

Research data are not shared.
