# Supplementary material for: CRISPR‐Enhanced Hydrogel Microparticles for Multiplexed Detection of Nucleic Acids
Source: Adv Sci (Weinh). 2023 Feb 1;10(10):2206872. doi: 10.1002/advs.202206872 (PMC10074104; doi:10.1002/advs.202206872)
Supplement: Supplementary file 1 — Supporting Information [file ADVS-10-2206872-s002.pdf]

ADVANCED SCIENCE
Open Access

Supporting Information

for *Adv. Sci.*, DOI 10.1002/advs.202206872

CRISPR-Enhanced Hydrogel Microparticles for Multiplexed Detection of Nucleic Acids

Yoon Ho Roh, Chang Yeol Lee, Sujin Lee, Hyunho Kim, Amy Ly, Cesar M. Castro, Jinwoo Cheon, Jae-Hyun Lee and Hakho Lee**

Supporting Information

CRISPR-enhanced hydrogel microparticles for multiplexed detection of nucleic acids

Yoon Ho Roh, Chang Yeol Lee, Sujin Lee, Hyunho Kim, Amy Ly, Cesar M. Castro, Jinwoo Cheon, Jae-Hyun Lee, and Hakho Lee**

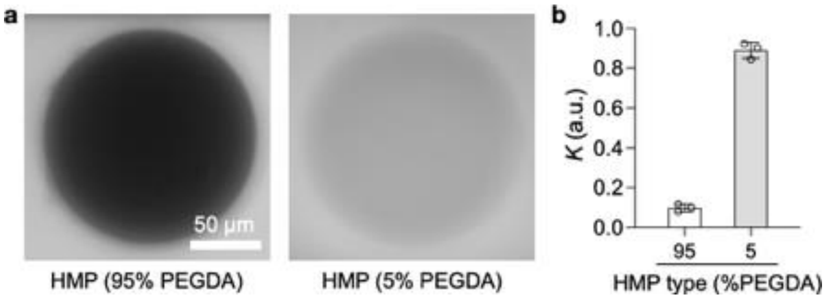

Figure S1. Characterization of HMP permeability. HMPs were synthesized at two different crosslinker concentrations (95% vs. 5% PEGDA) and incubated with FITC-dextran (150 kDa). **(a)** Fluorescent microscope images of HMPs. Non-porous HMPs (95% PEGDA) had negligible fluorescent signal, whereas FITC-dextran effectively diffused into the HMP crosslinked with 5% PEGDA. **(b)** The partition coefficient (*K*) was calculated as the intensity ratio between the HMP and the bulk solution. The K values were 0.098 for 95%-PEDGA HMPs and 0.889 for 5%-PEGDA HMPs. Data are displayed as mean ± sd from triplicate measurements. PEDGA, polyethylene glycol diacrylate; a.u., arbitrary unit.

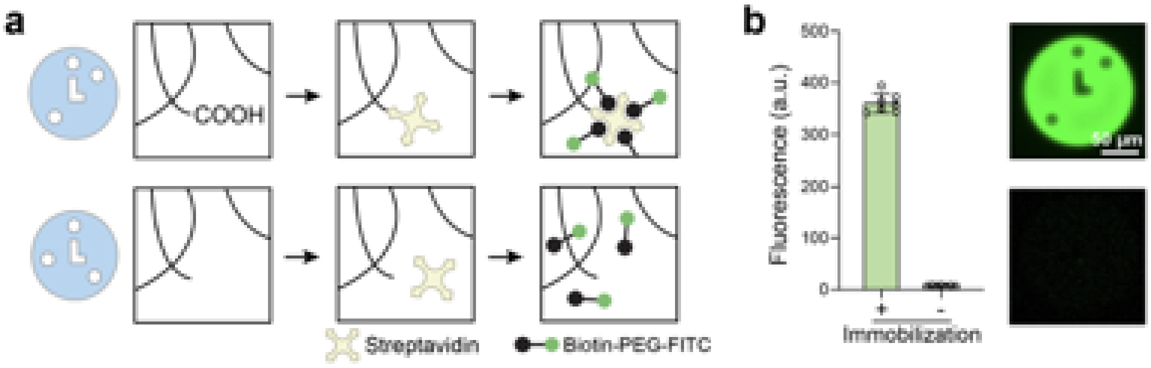

Figure S2. Importance of protein immobilization in HMPs. (a) Two types of HMPs, with and without a carboxyl (–COOH) group, were synthesized. Streptavidin was conjugated to COOH-containing HMPs. Both HMPs types were then incubated with fluorescent biotin, washed, and imaged. **(b)** Only streptavidin-conjugated HMPs maintained a high fluorescent signal after washing. Data are displayed as mean ± sd from seven HMPs. Scalebar, 50 µm.

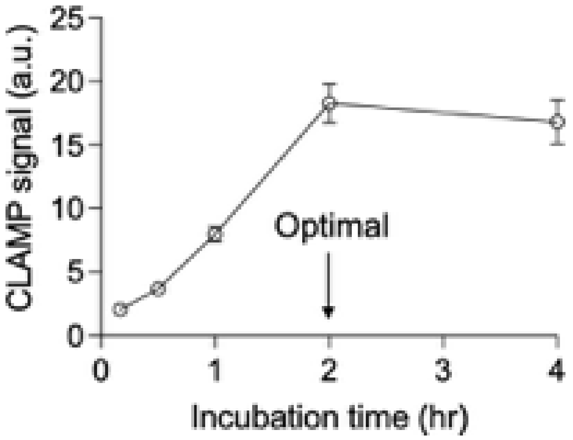

Figure S3. Optimization of Cas12a conjugation to HMPs. The conjugation time was varied between activated HMPs and Cas12a protein. The resulting HMPs were used for the CLAMP assay, and the fluorescent intensity was measured. The highest signal was observed with HMPs that were incubated with Cas12a for 2 hours. Data are displayed as mean ± sd from triplicate measurements.

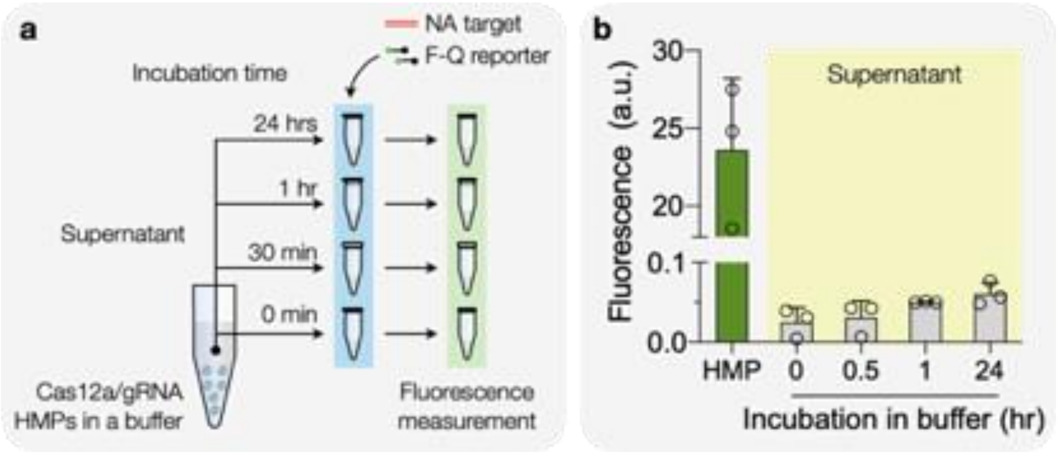

Figure S4. Stability of the Cas12a conjugation in HMPs. (a) Experiment scheme. Cas12a/gRNA HMPs were incubated in a buffer, and the solution-based Cas assays were performed using supernatant. **(b)** The observed fluorescent signals with supernatant (gray bars) remained low compared to the CLAMP signal from HMPs (green bar). The results indicate negligible Cas12a leaching from HMPs. Data are displayed as mean ± sd from technical triplicates.

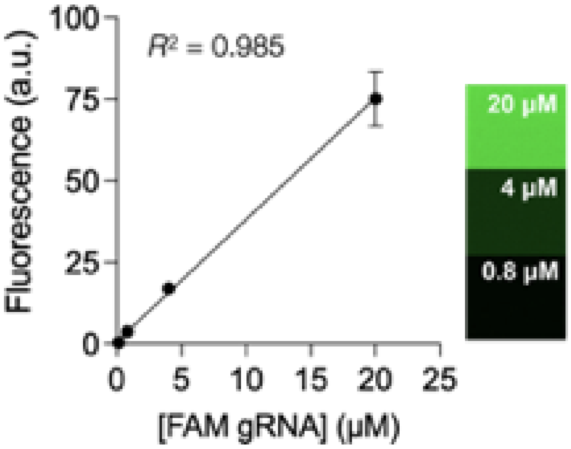

Figure S5. Calibration curve for FAM-gRNA solution inside the microfluidic device. FAM-gRNA of different concentrations was introduced to the microfluid device, and fluorescence images were taken (inset). From the measured data, a background-corrected calibration curve was generated. Data are displayed as mean ± sd from triplicate measurements.

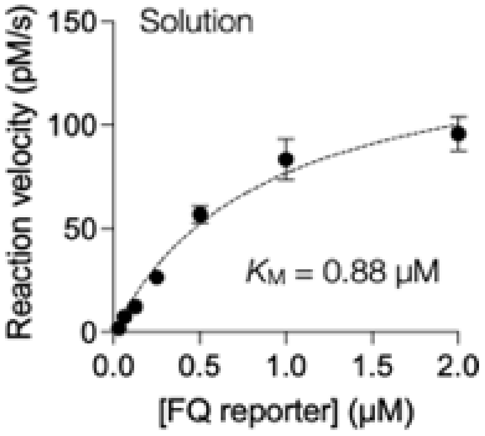

Figure S6. Enzyme kinetics in a solution-based Cas12a assay. Activated Cas12a/gRNA complexes were reacted with F-Q reporters of varying concentrations, and reaction velocities were measured. The dotted line indicates the fit to a Michaelis-Menten model. The enzyme turnover rate was k_{cat} = 0.29 $s^{-1,}$ and the Michalis-Menten constant was K_M = 0.88 µM. The calculated catalytic efficiency (k_{cat}/K_M) was 3.3 × 10^5 M^{-1} s^{-1}. Error bars indicate the standard deviation from three replicates.

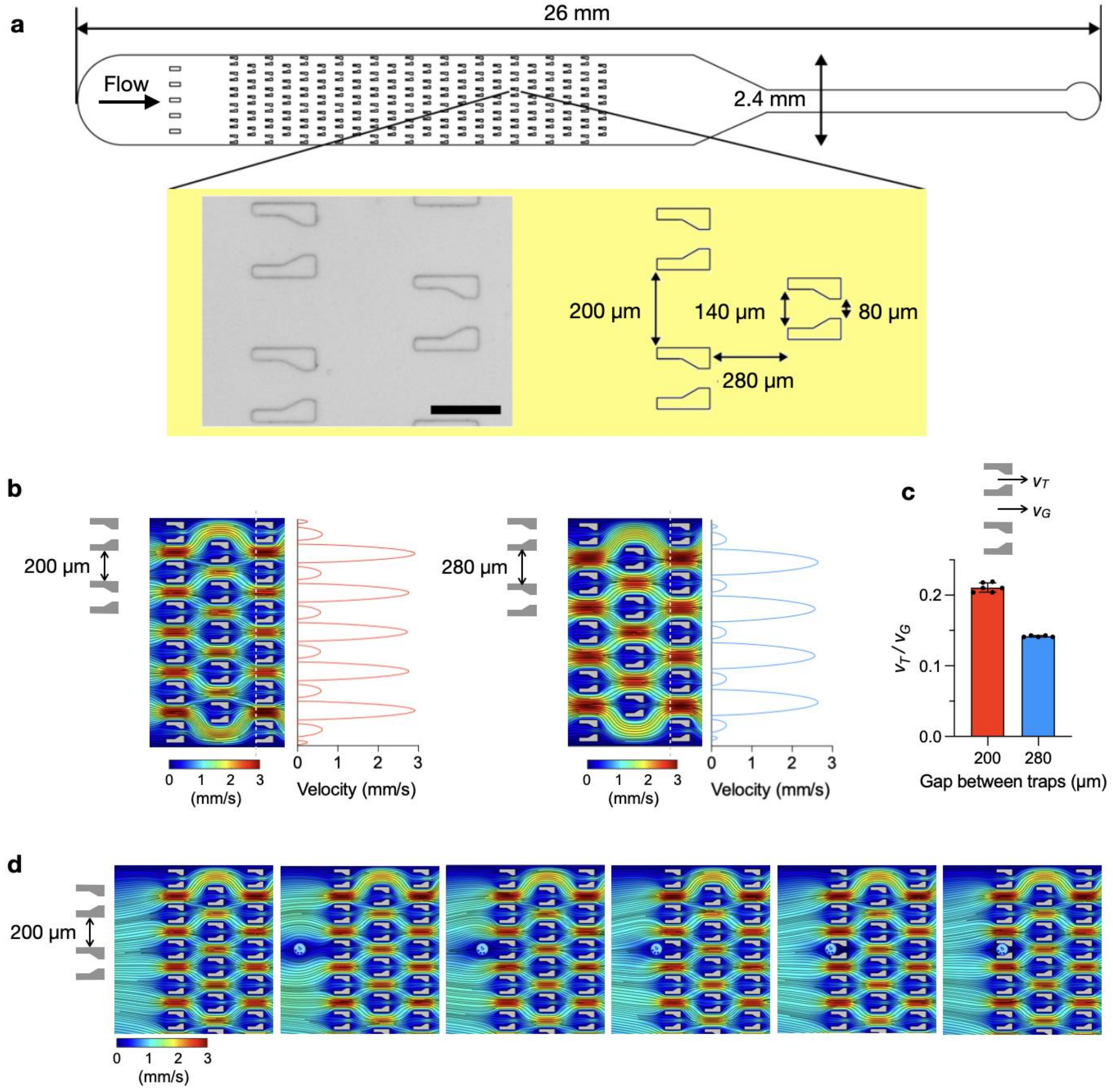

Figure S7. Microfluidic chip for HMP capture. (a) Final chip layout. The chip had 121 traps, each with an opening for a fluidic flow. Scale bar, 200 µm. **(b)** Fluidic simulation to optimize the trap arrangement. The gap between two adjacent traps was varied (200 µm, left; 280 µm, right), and the fluidic velocity and streamlines were simulated. The graph shows the velocity profile along the dotted line. **(c)** The fluidic speeds through the trap (v_T) and the gap (v_G) were compared. The design with a 200-µm gap had a higher v_T /v_G ratio than the 280-µm design, thereby improving the chance of HMP capture inside the trap. **(d)** A snapshot of fluidic flows during a single HMP capture.

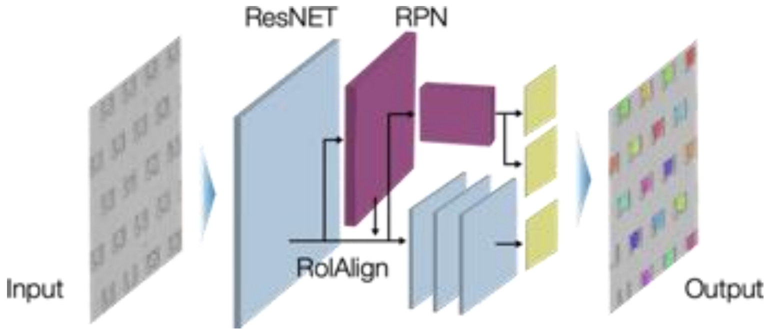

Figure S8. Mask R-CNN for HMP recognition. This neural network has three primary components: ResNet, region proposal networks (RPN), and RoIAlign. ResNet forms the backbone to extract features from an input image. RPN scans the feature map to detect candidate areas and refine candidates according to the confidence score. Then, RoIAlign pools the candidate areas into a feature map. Finally, two functional branches (class and box regression, mask generation) draw the prediction of the image.

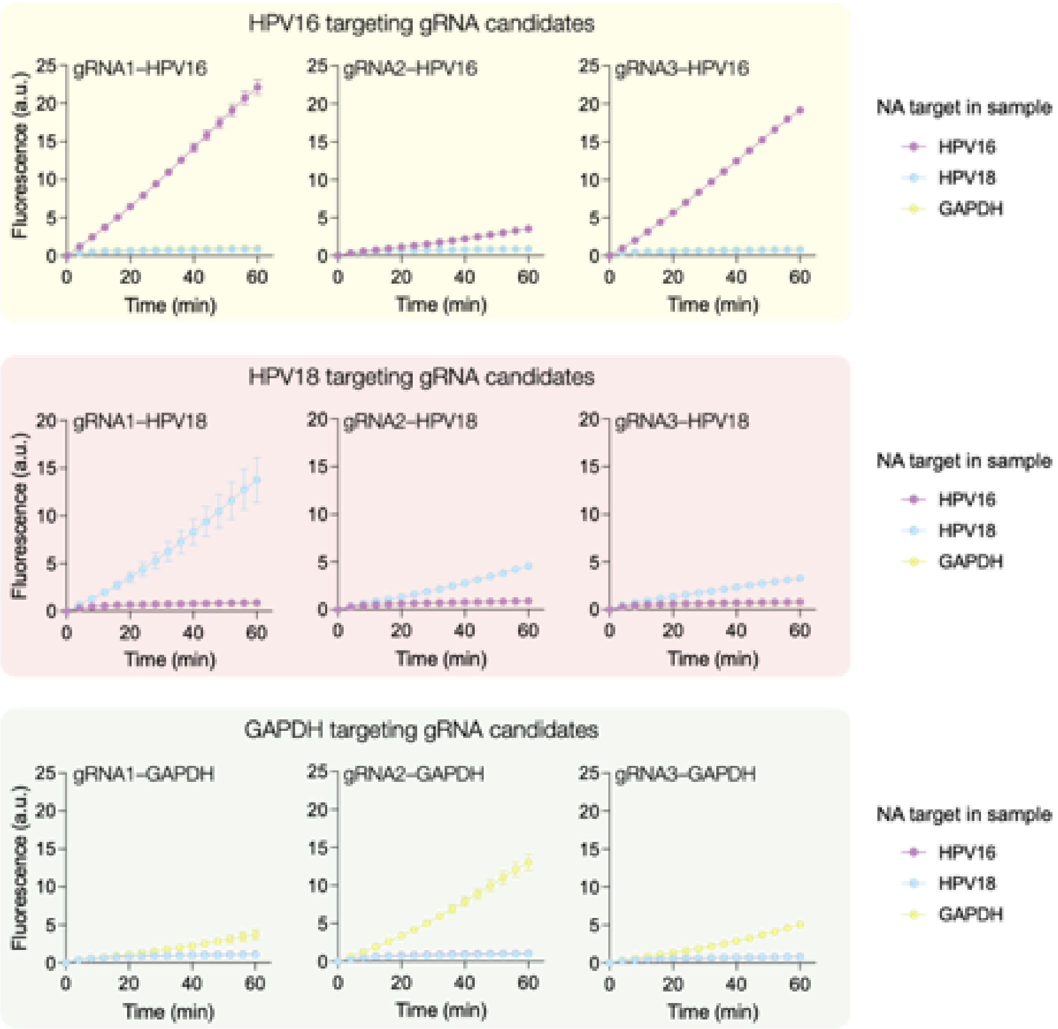

Figure S9. gRNA screening for one-pot multiplexing. For each NA target (HPV16, HPV18, or GAPDH), we tested three gRNA candidates for their specificity. Test samples contained either on-target or off-target NAs, and the assay was carried out in a solution-based Cas12a reaction. The first row shows the testing results of gRNAs specific to HPV16, the second row to HPV18, and the third row to GAPDH. The following were selected: gRNA1-HPV16, gRNA1-HPV18, and gRNA2-GAPDH. Error bars indicate the standard deviation from three replicates.

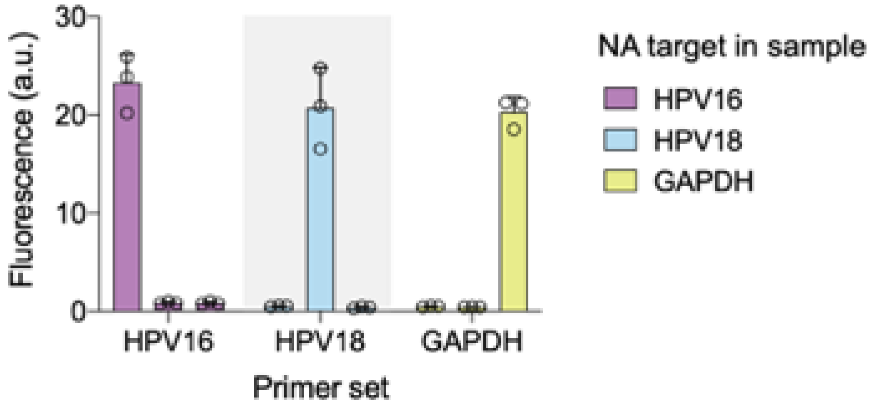

Figure S10. Validation of RPA primer specificity. Primer sets targeting either HPV16, HPV18, or GAPDH were reacted with on-target and off-target genes. Solution-based Cas12a assays confirmed the high specificity of designed primer sets. Data are displayed as mean ± sd from triplicate measurements.

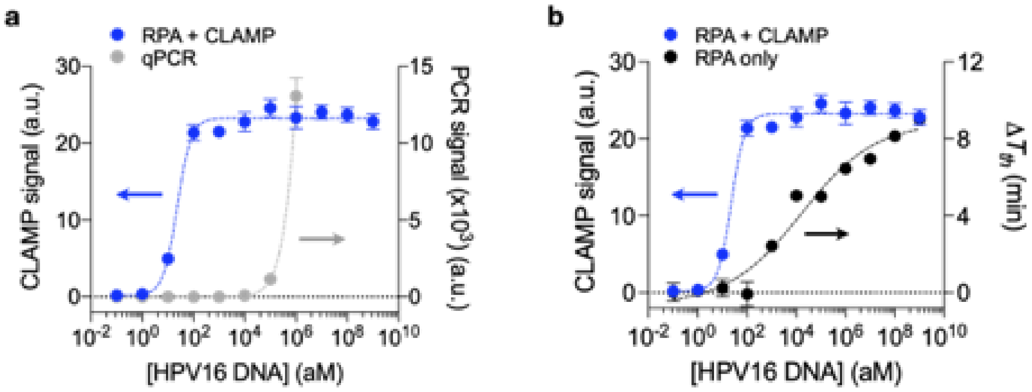

Figure S11. Comparison between CLAMP and other standard assays. (a) Serially diluted synthetic HPV16 DNA samples were analyzed by CLAMP and qPCR. CLAMP (2 aM; 1.2 copy/µL) outperformed qPCR (10 aM; 6 copy μL^{-1}) in sensitivity. Data are displayed as mean ± sd (*n* = 3). **(b)** RPA alone has a detection limit of 145 aM (87 copy μL^{-1}). Combing RPA with CLAMP lowered the limit down to 2 aM (1.2 copy μL^{-1}). For RPA, we used the threshold time (T_{th}) as a metric. At T_{th}, the analyte has fluorescence exceeding the threshold value. $\Delta T_{th} = T_{th}$ (HPV16 sample) – T_{th} (blank sample).

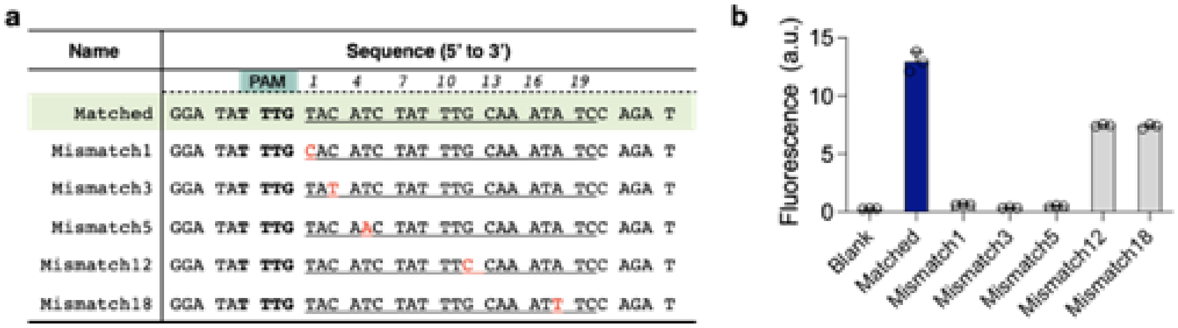

Figure S12. Specificity of CLAMP at the level of a single nucleotide. (a) Sequences of the matched and mismatched DNA targets. Within the gRNA recognition site (underlined), the position of a mismatching nucleotide (red) was varied from the PAM site (bold). **(b)** CLAMP assay results. When the mismatch was within the seeding region (~8 bp away from the PAM), the assay correctly differentiated the matched target from the mismatched one. [DNA] = 1 fM. Data are shown as mean ± sd from three samples (technical replicates).

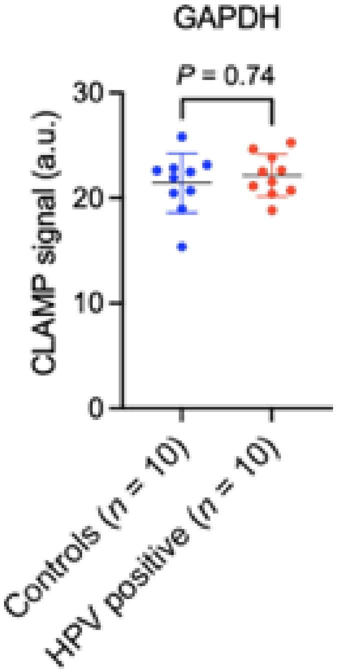

Figure S13. Comparison of GAPDH level in clinical samples. All samples passed quality control for cellularity. No significant difference in the GAPDH level was observed between non-cancer and high-risk samples (P = 0.74; unpaired, two-sided t-test).

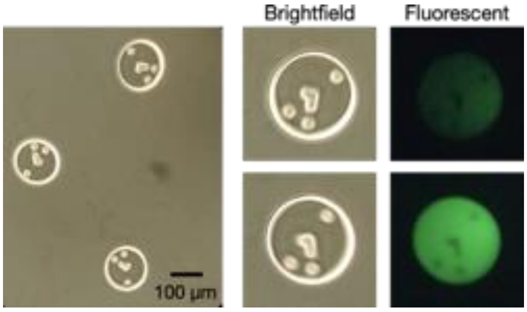

Figure S14. HMP imaging with a smartphone. Fluorescent HMPs were imaged by a smartphone (LG G6) camera. Brightfield imaging can be used to resolve HMP's identity code, while fluorescent imaging to quantify target DNA concentrations.

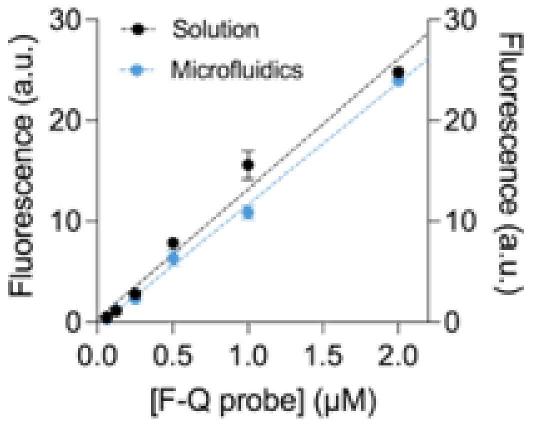

Figure S15. Calibration curves for fully-cleaved F-Q reporters. (a) Fluorescent intensities were measured in a solution-based Cas12 assay. **(b)** Fully cleaved F-Q reporters were injected into the microfluidic device, and fluorescent images were taken. Fluorescent intensities were obtained via imaging analysis. Error bars indicate standard deviations from three replicates.

Table S1. Performance comparison between CLAMP and other molecular tests.

System	Readout	Assay time (min)	LOD	Assay characteristics	Ref.
CRISPR/Cas12a-actuated sandwich sensor	SERS	40	6×10^{6} pM	• Singleplex<br>• No clinical validation<br>• Low sensitivity	1
RPA-CRISPR/Cas12a	LFA	30	10 aM	• Singleplex<br>• Subjective results	1
RPA-CRISPR/Cas12a (DETECTR)	Fluorescence	70	10 aM	• Singleplex	2
RPA-CRISPR/Cas12a	Fluorescence	35	17 aM	• Singleplex	3
CRISPR/Cas12a coupled with G-triplex	Fluorescence	140	0.1 aM	• Singleplex<br>• Precise temperature control for PCR	4
CRISPR/Cas12a-actuated spherical DNA reporter on gold nanoparticles	Fluorescence	120	10^{4} aM	• Singleplex<br>• No clinical validation<br>• Low sensitivity	5
Polymerase aptamer coupled with HRP	Color	180	10 amol	• Precise temperature control for asymmetric PCR<br>• Multiple detection sites for multiplex assay	6
CLAMP (current work)	Fluorescence	80	2 aM	• One-pot, multiplex assay	-

1. Su A, Liu Y, Cao X, Xu W, Liang C, Xu S (2022) A universal CRISPR/Cas12a-mediated AuNPs aggregation-based surface-enhanced Raman scattering (CRISPR/Cas-SERS) platform for virus gene detection. *Sens Actuators, B* 369:132295.
2. Chen JS, Ma E, Harrington LB, Da Costa M, Tian X, Palefsky JM, Doudna JA (2018) CRISPR-Cas12a target binding unleashes indiscriminate single-stranded DNase activity. *Science* 360:436-439.
3. Gong J, Zhang G, Wang W, Liang L, Li Q, Liu M, Xue L, Tang G (2021) A simple and rapid diagnostic method for 13 types of high-risk human papillomavirus (HR-HPV) detection using CRISPR-Cas12a technology. *Sci Rep* 11:12800.
4. Li T, Hu R, Xia J, Xu Z, Chen D, Xi J, Liu BF, Zhu J, Li Y, Yang Y, Liu M (2021) G-triplex: A new type of CRISPR-Cas12a reporter enabling highly sensitive nucleic acid detection. *Biosens Bioelectron* 187:113292.
5. Fu X, Shi Y, Peng F, Zhou M, Yin Y, Tan Y, Chen M, Yin X, Ke G, Zhang XB (2021) Exploring the Trans-Cleavage Activity of CRISPR/Cas12a on Gold Nanoparticles for Stable and Sensitive Biosensing. *Anal Chem* 93:4967-4974.
6. Ho NRY, Lim GS, Sundah NR, Lim D, Loh TP, Shao H (2018) Visual and modular detection of pathogen nucleic acids with enzyme-DNA molecular complexes. *Nat Commun* 9:3238.

Table S2. Oligonucleotide sequences used in this work.

Assay	Target	Probe	Sequence (5'→ 3')
RPA	HPV16	Forward primer	CAC AGT TAT TCA GGA TGG TGA TAT GGT TCA T
		Reverse primer	CAT ATG GTT CTG ACA CCA TTT TAA TAT AAT C
	HPV18	Forward primer	TTG CTG GCA TAA TCA ATT ATT TGT TAC TGT G
		Reverse primer	AAC ATG TCT GCT ATA CTG CTT AAA TTT GGT
	GAPDH	Forward primer	ACG GAT TTG GTC GTA TTG GGC GCC TGG TCA C
		Reverse primer	TTG ATG ACA AGC TTC CCG TTC TCA GCC TTG A
CRISPR reaction	HPV16	16-gRNA 1	UAA UUU CUA CUA AGU GUA GAU UAC AUC UAU UUG CAA AUA UC
		16-gRNA 2	UAA UUU CUA CUA AGU GUA GAU UUA CUG UUG UUG AUA CUA CA
		16-gRNA 3	UAA UUU CUA CUA AGU GUA GAU AGG AGU ACC UAC GAC AUG GG
	HPV18	18-gRNA 1	UAA UUU CUA CUA AGU GUA GAU UUA CUG UGG UAG AUA CCA CU
		18-gRNA 2	UAA UUU CUA CUA AGU GUA GAU ACA AUA UGU GCU UCU ACA CA
		18-gRNA 3	UAA UUU CUA CUA AGU GUA GAU AGC AGU AUA GCA GAC AUG UU
	GAPDH	GAPDH-gRNA 1	UAA UUU CUA CUA AGU GUA GAU GUC GUA UUG GGC GCC UGG UC
		GAPDH-gRNA 2	UAA UUU CUA CUA AGU GUA GAU ACU CUG GUA AAG UGG AUA UU
		GAPDH-gRNA 3	UAA UUU CUA CUA AGU GUA GAU CAU GUU CCA AUA UGA UUC CA
		Reporter	FAM-TTATT-BHQ1

Table S3. Critical parameters used in ML training.

Variable	Setting
Backbone	ResNet101
Images per GPU	1
Learning momentum	0.9
Learning rate	0.01, 0.001, 0.0001
Steps per epoch	100
Validation steps	50
Weight decay	0.0001
RPN anchor scales	(32, 64, 128, 256, 512)
RPN anchor ratios	[0.5, 1, 2]
